# A New Fracture Detection Algorithm of Low Amplitude Acoustic Emission Signal Based on Kalman Filter-Ripple Voltage

**DOI:** 10.3390/s21124247

**Published:** 2021-06-21

**Authors:** Seong-Min Jeong, Seokmoo Hong, Jong-Seok Oh

**Affiliations:** 1 Department of Future Convergence Engineering, Kongju National University, Cheonan 31080, Korea; tjdals7699@gmail.com; 2 Department of Future Automotive Engineering, Kongju National University, Cheonan 31080, Korea

**Keywords:** Kalman filter, ripple noise, acoustic emission, fracture detection, press process

## Abstract

In this study, an acoustic emission (AE) sensor was utilized to predict fractures that occur in a product during the sheet metal forming process. An AE activity was analyzed, presuming that AE occurs when plastic deformation and fracturing of metallic materials occur. For the analysis, a threshold voltage is set to distinguish the AE signal from the ripple voltage signal and noise. If the amplitude of the AE signal is small, it is difficult to distinguish the AE signal from the ripple voltage signal and the noise signal. Hence, there is a limitation in predicting fractures using the AE sensor. To overcome this limitation, the Kalman filter was used in this study to remove the ripple voltage signal and noise signal and then analyze the activity. However, it was difficult to filter out the ripple voltage signal using a conventional low-pass filter or Kalman filter because the ripple voltage signal is a high-frequency component governed by the switch-mode of the power supply. Therefore, a Kalman filter that has a low Kalman gain was designed to extract only the ripple voltage signal. Based on the KF-RV algorithm, the measured ripple voltage and noise signal were reduced by 97.3% on average. Subsequently, the AE signal was extracted appropriately using the difference between the measured value and the extracted ripple voltage signal. The activity of the extracted AE signal was analyzed using the ring-down count among various AE parameters to determine if there was a fracture in the test specimen.

## 1. Introduction

The occurrences of defects in sheet metal formation can cause huge losses in productivity. Furthermore, if sheet metal forming tasks are performed without detecting defects in the die, the die itself could be damaged. Therefore, to develop a system for identifying defects in sheet metal forming, several studies have been conducted in recent years to develop a defect detection system that utilizes a new inspection method based on acoustic emission [1,2,3].

Acoustic emission (AE) refers to an acoustic signal that is generated when the strain energy that is formed locally in a solid material is rapidly released [4]. AE is used for detecting predicted fractures in bearings [5], gearboxes [6], pressure vessels [7], and pipes [8,9] at many sites in the industry. Moreover, an AE signal is generated when an object is plastically deformed or fractured. When a metallic material exceeds the maximum load and is close to fracturing, AE occurs frequently. Hence, AE is widely applied for evaluating the mechanical properties of materials [10,11,12,13,14].

**Figure 1:**
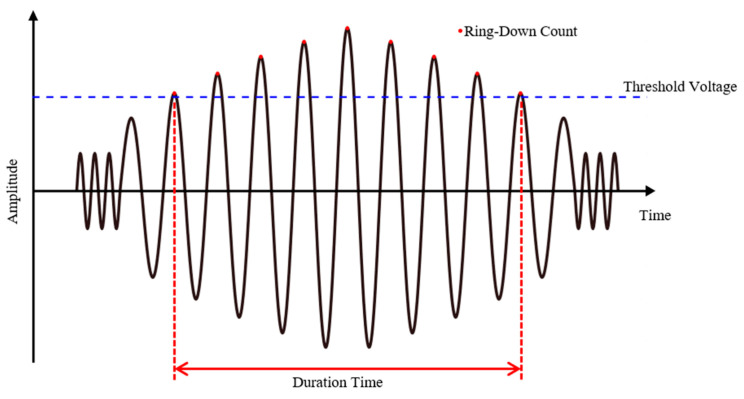
AE parameter.

The analysis method of such an AE signal can be largely divided into waveform analysis related to the activity and the shape of the signal [10,15]. The AE activity analysis method evaluates various parameters such as the number of events, ring-down count, and maximum amplitude. The analysis method is directly related to the size and frequency of the source of the AE. In Figure 1, the ring-down count refers to the number of AE vibrations that cross the detection threshold. On the contrary, the method based on waveform analysis compares the waveform shapes of the AE signals. The parameters utilized in this method include duration time and frequency spectrum. Duration time refers to the time elapsed between the time the wave height first exceeds the threshold voltage and the time the wave height is less than the threshold voltage from the same AE event.

To apply the AE signal analysis method to real manufacturing processes, it is essential to distinguish the noise or ripple voltage signal from the AE signal [16,17,18,19]. Hence, a trigger algorithm using the threshold voltage value [20,21,22,23] and a low-pass filter that removes high-frequency noise [24] have been widely applied. The threshold voltage is a voltage that recognizes the AE signal. The value of the threshold voltage should be set considering the magnitude of the surrounding noise and the magnitude of the AE signal.

**Figure 2:**
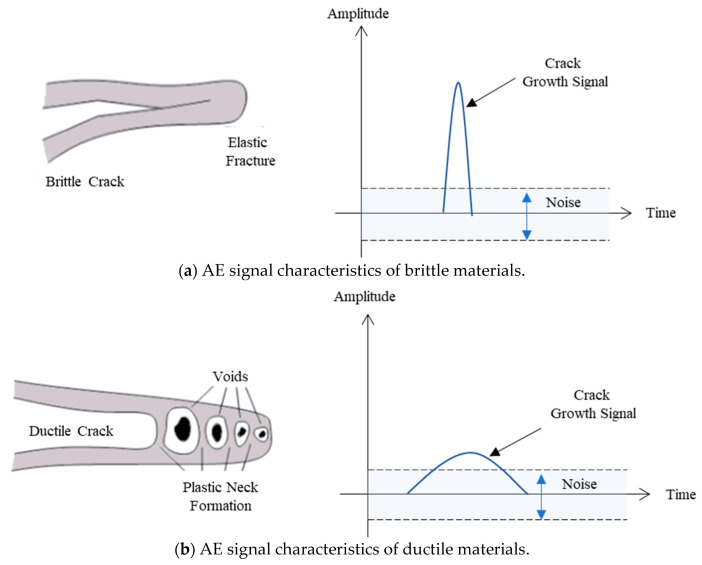
AE signal amplitude according to fracture characteristics.

However, even these suggested algorithms have considerable difficulty detecting an AE signal because the AE signal for ductile materials has a very high signal-to-noise ratio (SNR) at factories where sheet metal forming is performed [4,25]. As shown in Figure 2, the AE signal amplitude is closely related to the crack growth speed. Due to the crack growth speed of brittle materials being very high, as shown in Figure 2a, the signal amplitude is large. However, the fracture toughness of ductile materials is very large, as shown in Figure 2b. The generation and growth of the crack occur very slowly, and the signal amplitude is relatively small. Hence, the application of the AE sensor for ductile materials was limited by the low amplitude signal and inevitable noise. Previous studies related to the press process show good results for little SNR, whereas the relatively poor detection performance was obtained from the material with high SNR [1,2,3].

Moreover, power must be supplied to actuate the AE sensor, and the ripple voltage signal of the power supply is transmitted as the output through various paths. In a switch-mode power supply, a switching regulator receives power from an alternating current (AC) power source and converts it to direct current (DC) to supply the power. Here, the AC is not entirely converted to DC, and the remaining AC signal is known as the ripple voltage signal [26,27]. The ripple voltage signal follows the driving frequency of the switching regulator, and this frequency is higher than the frequency of the AE signal. Hence, it is difficult to cancel the ripple voltage signal using a low-pass filter. The ripple voltage signal from the power supply is a factor that further restricts the use of AE sensors that have a low voltage signal value. To distinguish the AE signal from ripple voltage and noise signals, a deep learning algorithm such as the convolutional neural network (CNN) method can be applied. From the results in previous studies [28,29,30], it has been proved that the deep learning method shows a good detection performance. However, since the CNN method requires a frequency domain image file such as spectrogram or scalogram, it cannot detect the failure of the system in real-time. It is very important to detect the occurrences of defects in the manufacturing field in real-time and even early. Therefore, in this study, a Kalman filter was applied with a low Kalman gain to an algorithm that processes AE signals containing noise and ripple voltage signals to predict fractures in ductile materials during sheet metal forming.

Although the AE sensor has been applied to many applications in the manufacturing process, it has not yet been applied to the metal sheet forming process for a ductile material. The advantage of the proposed method is to detect the defect of the material, which has a very high AE signal-to-noise ratio in real-time. The noise makes it difficult to detect the fracture of the specimen by using the AE sensor. Moreover, the ripple voltage is unavoidable for the measurement system with a power supply. Many previous studies focus on materials that have a low AE signal-to-noise ratio. Therefore, the primary purpose of this manuscript is to explore the potentials of KF-RV as a fracture detection method for the metal sheet forming process. Therefore, in this study, a Kalman filter with a low Kalman gain was applied to the AE signal processing algorithm to predict fractures in ductile materials during sheet metal forming. In order to prove this, the authors present the simulation results for virtually synthesized signals. The proposed estimation algorithm and simulation results are presented in the section: Design of Kalman Filter-Ripple Voltage. Next, the sheet metal forming process is experimentally conducted by using a pressing device, and the raw sensor data is measured, as described in the section: Sheet Metal Forming Experiment. Finally, in the section: AE Detection Method and Discussion, it has been confirmed that the AE detection method can determine the accurate time at which a defect occurs in the product during the sheet metal forming process.

## 2. Design of Kalman Filter-Ripple Voltage

In this study, the Kalman filter was applied to the signal-processing algorithm of an AE sensor. The Kalman filter is divided into two stages—estimating the current state and estimating the Kalman gain using the measured value. In the current state estimation stage, the previous value is used to predict the next value, and the error covariance is used to estimate the value for the subsequent period. In the Kalman gain estimation stage, a new value is included in the previous value to obtain the supplemented subsequent value. The discrete-time state-space model of the AE sensor is defined as follows for the Kalman filter design [31,32,33,34].

(1) $$\begin{array}{l} x\left(k+1\right)=A_{d}x\left(k\right)+w\left(k\right)\\ z\left(k\right)=Hx\left(k\right)+\nu \left(k\right)\\ A_{d}=\left[\begin{array}{cc} 1 & T_{a}\\ 0 & 1 \end{array}\right],H=\left[\begin{array}{cc} 1 & 0 \end{array}\right],x\left(k\right)={\left[\begin{array}{cc} a\left(k\right) & \dot{a}\left(k\right) \end{array}\right]}^{T} \end{array}$$

where, *z* is the measured value from the sensor, **A***_d_* is the system matrix of the discrete system, and **H** is the measurement matrix. w and v denote the noise matrices of the system and measurement model, respectively, and they are assumed to be Gaussian noises. **x**(*k*) is the state vector matrix. *T_a_* is the sampling time. In this study, *T_a_* is assigned a value of 500 kHz. The subscript *d* denotes the discrete-time, and the Kalman filter is designed based on the discrete-time measurement model, as shown below.

**Figure 3:**
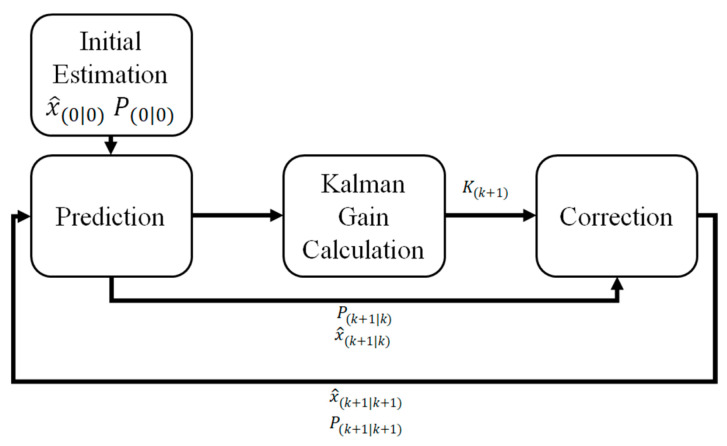
Recursive structure algorithm of the Kalman filter.

- Prediction Stage
  (2) $$\begin{array}{l} \hat{x}\left(k+1|k\right)=A_{d}\hat{x}\left(k|k\right)\\ P\left(k+1|k\right)=A_{d}P\left(k|k\right)A_{d}{}^{T}+Q_{d} \end{array}$$
- Kalman gain calculation stage
  (3) $$K\left(k+1\right)=P\left(k+1|k\right)H^{T}{\left[HP\left(k+1|k\right)H^{T}+R_{d}\right]}^{-1}$$
- Correction stage
  (4) $$\begin{array}{l} \hat{x}\left(k+1|k+1\right)=\hat{x}\left(k+1|k\right)+K\left(k+1\right)\left[z\left(k+1\right)-H\hat{x}\left(k+1|k\right)\right]\\ \;\;\;\;\;\;\;=K\left(k+1\right)z\left(k+1\right)+\left(1-K\left(k+1\right)H\right)\hat{x}\left(k+1|k\right) \end{array}$$
  P(k+1|k+1)=[I−K(k+1)H]P(k+1|k)
  where, **Q***_d_* is the covariance matrix of system noise, **R***_d_* is the covariance matrix of measurement noise, and **K** is the Kalman gain. $\hat{x}\left(k+1|k\right)$ is the pre-estimated state vector matrix and P(k+1|k) is the pre-estimated error covariance matrix. These matrices are used to estimate the state vector matrix $\hat{x}\left(k+1|k+1\right)$ and error covariance matrix P(k+1|k+1) in the next stage. Moreover, **Q***_d_*, which was used to estimate the error covariance, is a system model parameter. As the value of **Q***_d_* increases, the effect of the measured value increases. Likewise, the effect of the measured value decreases as the value of **Q***_d_* decreases. In order to simplify the procedure, it is assumed as the product of identity matrix and constant, Q. Therefore, if the Kalman gain increases, the proportion of the measured value (*z*(*k* + 1)) increases. Similarly, if the Kalman gain decreases, the proportion of the predicted value ($\hat{x}\left(k+1\right)$) increases. This relationship can be seen from the equation for calculating the estimated value in the correction stage. The algorithm with a recursive structure, as shown in Figure 3, summarizes the various stages of the Kalman filter, as described previously.

**Figure 4:**
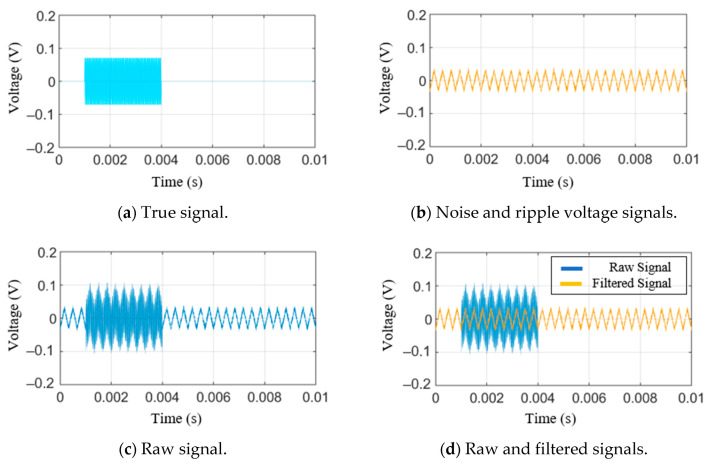
Simulation results of the classical Kalman filter.

A simulation was performed using virtual, synthesized signals to verify the filtering performance of the Kalman filter. As shown in Figure 4a, the signal is in the shape of a sine wave between 0.001 and 0.004 s. Beyond this period, the true signal has a value of zero. Signals other than the true signal include Gaussian noise and ripple voltage signals. Figure 4b shows a signal in the shape of a triangular wave. This signal represents a combined signal of the ripple voltage signal and Gaussian noise. Combining the true signal with the Gaussian noise and the ripple voltage signal generates the signal shown in Figure 4c. This signal was assumed to be the measured signal. The Kalman filter described earlier was applied to the artificially synthesized and measured signal, and the result is shown in Figure 4d. The blue line denotes 0.1 as the value of Q, and the red line denotes $1.0\times 10^{-5}$ as the value of Q. If the value of Q increases, the predicted value of the error covariance increases. When the predicted value of the error covariance increases, the Kalman gain increases accordingly, this result reflects the measured value to a greater degree. Therefore, as the value of Q decreases, only the values between 0.001 and 0.004 s, which is the interval within which the true signal has non-zero values, decrease while the values in the other intervals do not change. Thus, it was verified that the error due to the ripple voltage signal is very significant. The Kalman filter assumes that the noise is Gaussian. However, the ripple voltage signal is periodic, not a Gaussian noise. This discrepancy adversely affects the performance of predicting the true signal [34].

To solve this problem, a small Q value was used to minimize the Kalman gain. If the Kalman gain is decreased, the value of the system model becomes important to the resulting value of the Kalman filter. Moreover, it slows down the reflection of the measured value so that the ripple voltage signal can be derived appropriately. This effectively filters out the Gaussian noise, but the ripple voltage signal, which is a non-Gaussian noise, remains. Due to the signal being measured instantaneously having a very high frequency, it slows down the reflection of the estimated value. The ripple voltage signal extracted in this manner is subtracted from the raw signal to obtain the desired true signal. The value of Q was determined via the trial-error method. In order to obtain a better noise canceling performance, the value was set as $1.0\times 10^{-5}$ during repeated simulations. We denoted this algorithm as the Kalman filter-ripple voltage (KF-RV), and it is defined below:

(5) $$\begin{array}{l} \;\;\;\;\;\;\;\;\;z\left(k+1\right)-\hat{x}{\left(k+1|k+1\right)}_{KF-RV}\\ \;\;\;\;\;\;\;\;\;\;=z\left(k+1\right)-\hat{x}\left(k+1|k\right)+K\left(k+1\right)\left[z\left(k+1\right)-H\hat{x}\left(k+1|k\right)\right]\\ \;\;\;\;\;\;\;\;\;\;\;=\left(1-K\left(k+1\right)\right)z\left(k+1\right)+\left(1-K\left(k+1\right)H\right)\hat{x}\left(k+1|k\right) \end{array}$$

**Figure 5:**
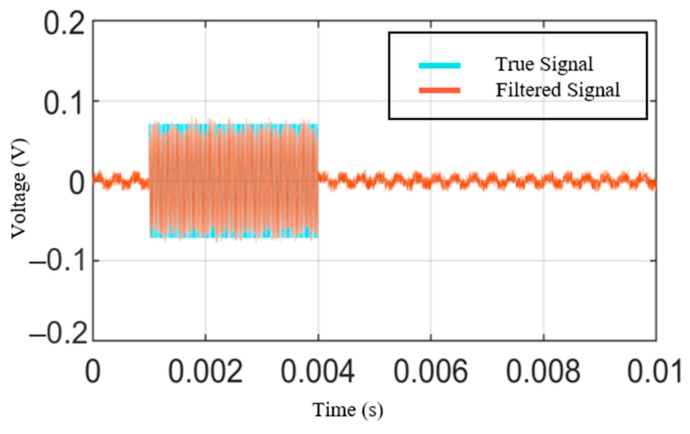
Simulation results of KF-RV.

The result of applying this filter to the virtual signal is shown in Figure 5. The light blue line represents the true signal, and the red line represents the signal to which the proposed KF-RV is applied. The error rate can be calculated as *e_rms_* and *e_max_* [26]. These values are obtained by dividing the average of the difference between the measurement and the estimation by the root mean square (RMS) of the measurement and the maximum value of the measurement, respectively. The error rate for the virtual signal was calculated to verify the performance of the proposed algorithm. The results are shown in Table 1, and it was confirmed that the proposed algorithm has a much superior filtering effect on the ripple voltage signal than the classical Kalman filter.

(6) $$e_{rms}=\frac{1}{X_{r,meas,rms}}\sum_{i=1}^{N}\frac{\left|X_{r,meas}(i)-X_{r,est}(i)\right|}{N}$$

(7) $$e_{max}=\frac{1}{X_{r,meas,max}}\sum_{i=1}^{N}\frac{\left|X_{r,meas}(i)-X_{r,est}(i)\right|}{N}$$

## 3. Sheet Metal Forming Experiment

**Figure 6:**
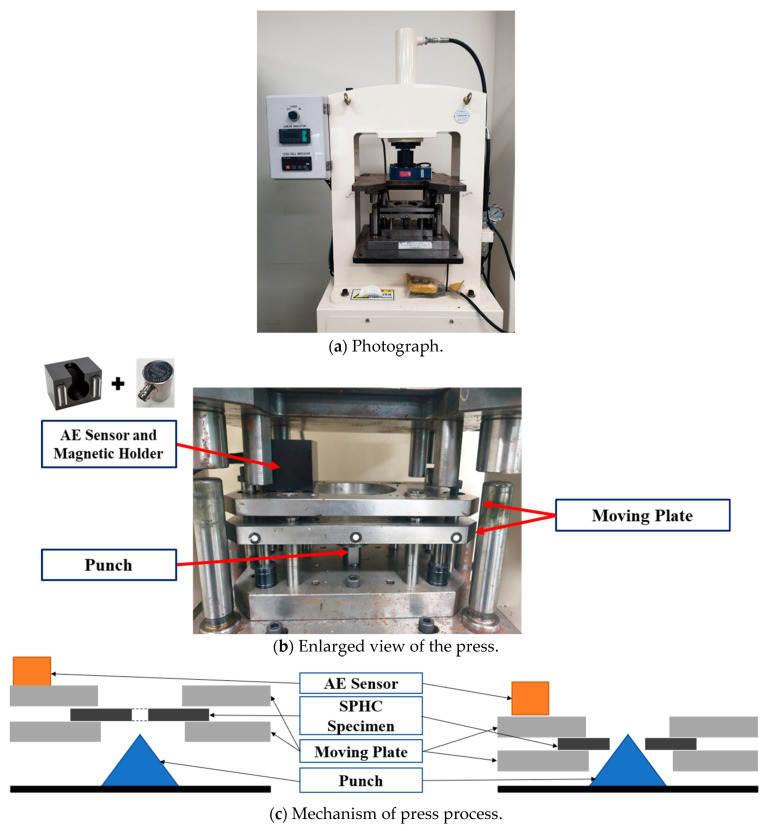
Hole expansion test using AE sensor.

The usefulness of this study was verified through the hole expansion test conducted during the sheet metal forming process. The press equipment used in the experiment is shown in Figure 6a. It consists of a pressure gauge, a press body, an indicator and a load cell and has a maximum load of 5000 N and a maximum stroke of 225 mm. The hole expansion specimen, shown in Figure 6, is placed on a moving plate, and both ends of the specimen are fixed. While the moving plate of the press is lowered, the specimen undergoes plastic deformation, and the hole expands. When the moving plate reaches the bottom dead center, it stops. AE signals are collected over the whole stroke range, and results are analyzed. Figure 6b,c show, the schematic diagrams before and after the hole expansion test. The AE sensor could not be attached directly to the workpiece due to the characteristics of sheet metal forming. Therefore, this device was fabricated in such a way that the sensor could be attached to and detached from the upper moving plate that is closest to the workpiece. Moreover, the sensor was fixed to one side of the moving plate to ensure that it is not affected by the vibrations of the operating press. The test has been conducted in a semi-outdoor laboratory and indoor laboratory. The semi-outdoor and indoor laboratory temperatures are 18 °C and 20 °C, respectively. Furthermore, the humidity of semi-outdoor and indoor laboratory humidity is 40–60%.

It is generally known that the main frequency range of fracture for low-carbon steel is from 60 to 500 kHz [35]. In order to exactly analyze the frequency range of commercial-quality hot-rolled steel (SPHC), the AE signal during the hole expansion test was measured and analyzed by using the AE sensor for the broadband frequency range. From the frequency analysis of preliminary test results, it is confirmed that the maximum peak frequency of the AE signal for SPHC is 60 kHz. Since resonant AE sensors have high sensitivity and narrow frequency bandwidth, the resonant type of AE sensor (Physical Acoustics Corp., West Windsor Township, NJ, USA, R6I-AST) is selected to detect AE signals with a low amplitude [36]. The specifications of the AE sensor are listed in Table 2. The peak sensitivity (V/μbar) is −23 dB, and the operating frequency region is 40–100 kHz. The AE sensor used in the AE measurements is made of piezoelectric materials such as lead zirconate titanate (PbZrxTi1-XO3, PZT). It has been generally known that PZT can convert structural vibration energy into electrical energy. The AE signal is transferred from specimen to sensor, and then the deformation of PZT occurred, and the electrical energy is generated. As shown in Figure 6b, the AE sensor is mounted to the moving plate by using a magnetic holder. The magnetic holder is electrically isolated to the AE sensor. The triboelectric effect, which is a type of contact electrification between dissimilar materials, can make additional noise. In order to reduce the triboelectric noise, a double-shielded BNC cable is used to prevent the noise. Furthermore, any movements such as twisting or transient impact are restricted during the test. AE signals are converted to digital signals and transferred to each other via an A/D, D/A board, and a data acquisition board (NI Corp., Austin, TX, USA, USB-6341). For more information on the data acquisition board, please refer to reference [37].

The die used in the experiment is used for the hole expansion test. As illustrated in Figure 6, the test specimen was lowered onto a punch, which has a shape pointed to the top and is fixed to the lower part, to expand the hole in the test specimen. The characteristics of the deformation changed according to the size of the hole and the thickness of the test specimen. In the case of a thin plate and a large hole, the hole expanded without fracture. However, when the hole size was relatively small, a fracture occurred while the hole was expanding. The test specimen used was SPHC, and the thickness of the plate was 2 mm. The modulus of elasticity was 210 MPa, the yield stress was 257 MPa, and the tensile strength was 270 MPa. Furthermore, SPHC that has an elongation of 31% is a classic ductile material. The SPHC test specimens had a thickness of 2 mm before the hole expansion test, and the test was conducted using the test specimens with a 4 mm hole drilled in the center. Four identical test specimens were prepared, and the test was performed two times at different sites. The same press equipment was used for all four test specimens. Tests #1 and #2 were conducted in a laboratory exposed to the outside environment, whereas tests #3 and #4 were conducted in a laboratory environment.

**Figure 7:**
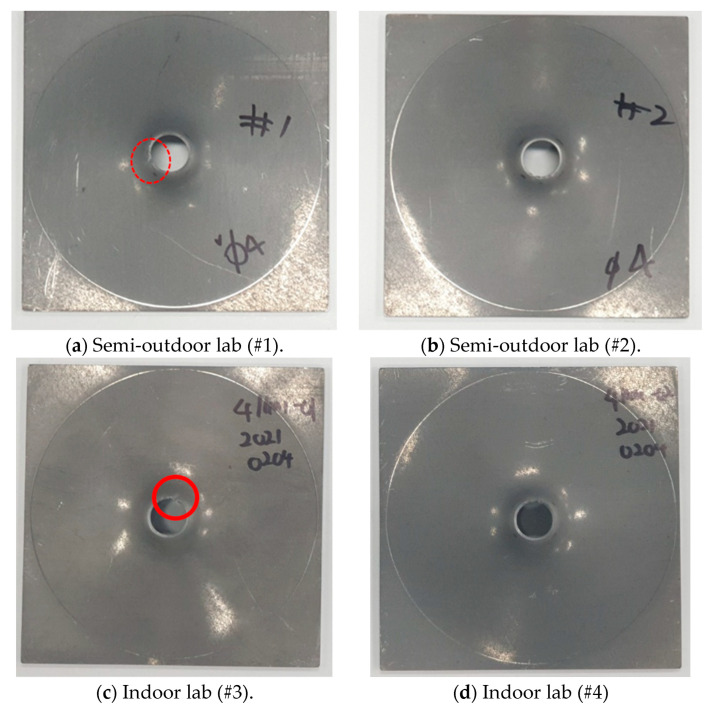
Images of test specimens after the hole expansion test.

Figure 7 shows the test specimens after the hole expansion test; the test specimens have holes of the same size. In Figure 7b,d, plastic deformation occurred, in which only the size of the hole was expanded. However, when the diameter of the hole became much larger than the initial diameter, a fracture occurred, as indicated by the red circle in Figure 7a,c. The deformation type of the specimen is determined according to yield strain, ultimate tensile strength, necking strain, and fracture strain. The strain values are measured by DIC (Digital Image Correlation). For more details of strain measurement, please refer to our previous study [38]. The difference is caused by various factors, such as minuscule differences in the thickness of the test specimens, as well as the hole-processing precision and alignment. This study was conducted to distinguish the occurrence of fracture in the test specimens due to these causes using the AE. 

**Figure 8:**
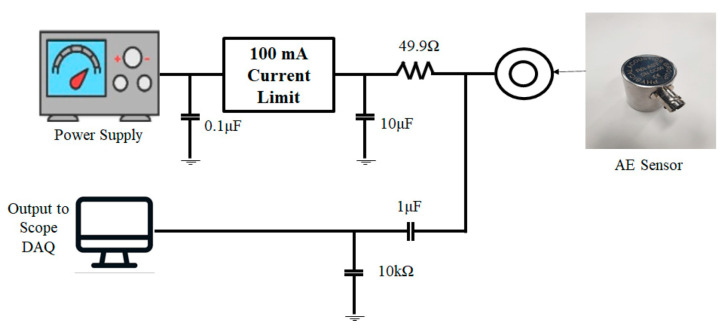
Driving circuit for AE sensor.

During the pressing process, the pre-amplifier receives the AE signal generated from the workpiece. The pre-amplifier then amplifies the signal and transmits it to the measuring device. Figure 8 shows the circuit diagram of the AE sensor and the DAQ device [39]. As shown in the circuit diagram, a power supply that can provide a voltage less than or equal to 28 V and a current less than or equal to 100 mA and a separate current limiting circuit are needed for the AE sensor to operate properly.

The AE signal is generated in a very short time. However, because the sampling time is also very short, the data volume is vast. Hence, an appropriate threshold voltage is set for a typically processed signal, as described in the introduction. It is common to analyze the aforementioned AE parameters based on the threshold voltage [10,11,12,13,14,15,16,17,18,19,20,21,22,23,24].

**Figure 9:**
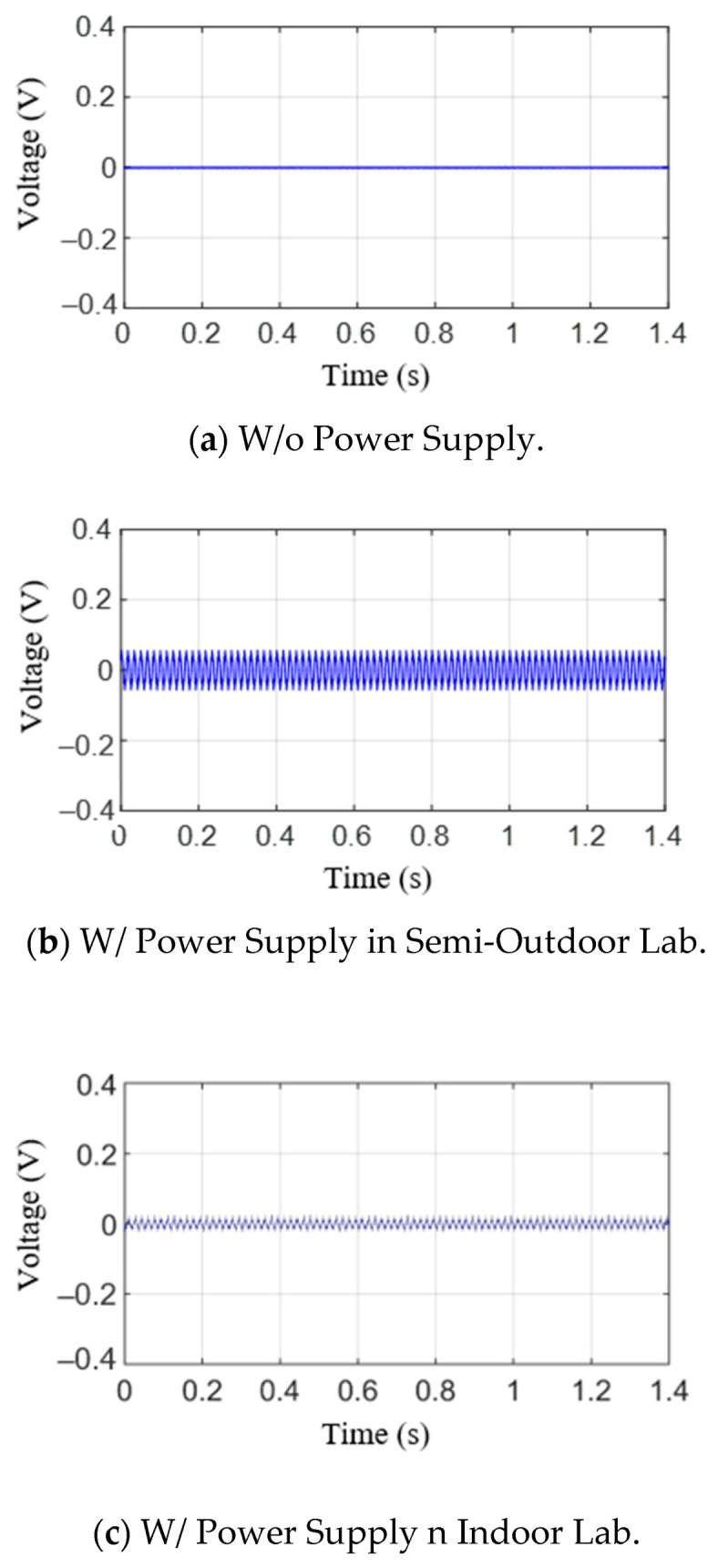
AE raw signal without press process.

Figure 9 illustrates the measurement results when the press is in a non-operative state during the hole expansion test. However, because the press was not operating, only the noise and ripple voltage signals were measured. Figure 9a shows the measured results without power supply input. The value is zero, but the measured results with power supply input are not zero, as shown in Figure 9b,c. The peak-to-peak values of the semi-outdoor lab and indoor labs are 0.1010 V and 0.0493 V, respectively. In other words, the maximum values are 0.0506 and 0.0246 V, respectively; the minimum values are −0.0504 and −0.0247 V, respectively. From Figure 9, the ripple voltage signal is a result of the power supply. The signals are repeatedly measured three times, and the peak amplitudes of the signal are almost the same. 

**Figure 10:**
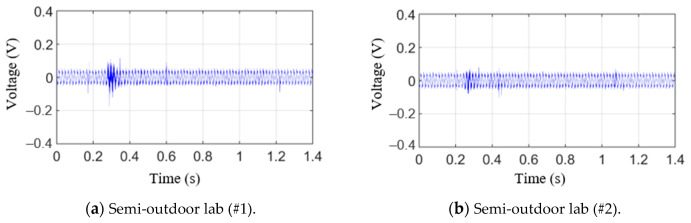

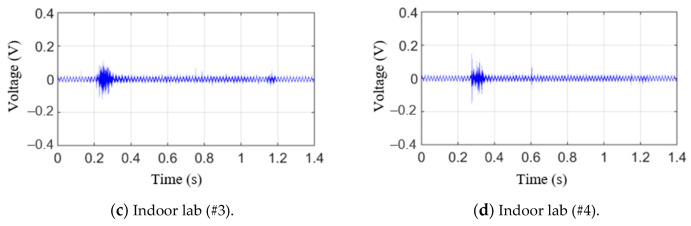
AE raw signal with press process.

Figure 10 shows the raw AE signals for testing materials in the time domain. The signals, as shown in Figure 10a,b, were obtained in a laboratory exposed to the outside environment, and the signals shown in Figure 10c,d were obtained in a laboratory environment. From these figures, it can be seen that the generated AE signal is not much larger than the noise and ripple voltage signals, as illustrated in Figure 9. The positive threshold voltage was set to 0.07 V based on the measured results to derive the aforementioned AE parameters. Here, 0.07 V is slightly larger than the noise level. Based on this value, previously described AE parameters were obtained, and the results are shown in Table 3. As can be seen from the results, it was difficult to uncover the correlation between the presence or absence of a fracture and the parameters, such as ring-down count, duration time, and peak amplitude. First, the ring-down counts for #3 and #4 were similar, but the presence or absence of a fracture for #3 and #4 was different. Moreover, the peak amplitudes were similar for #1 and #4 and #2 and #3. However, the presence or absence of a fracture was again different. Lastly, #2 and #1 had the longest and shortest duration time, respectively. Moreover, no fractures were observed in either #2 or #4. Furthermore, because the threshold voltage was set high due to a large ripple voltage signal, we determined that it was difficult to analyze the correlation between the presence or absence of a fracture and the parameters. 

**Figure 11:**
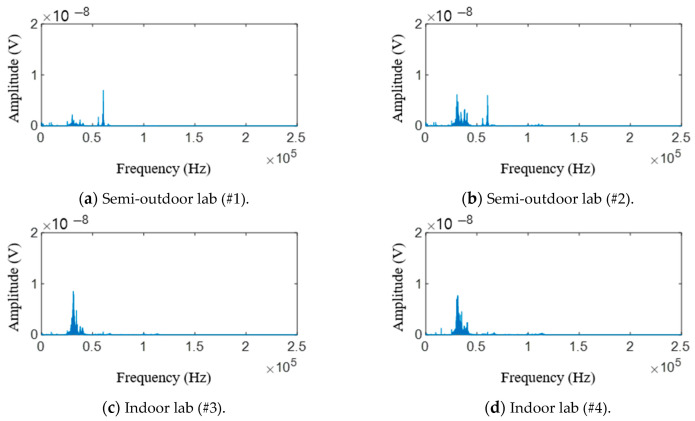
Result of fast Fourier transform applied to AE filtered signal.

**Figure 12:**
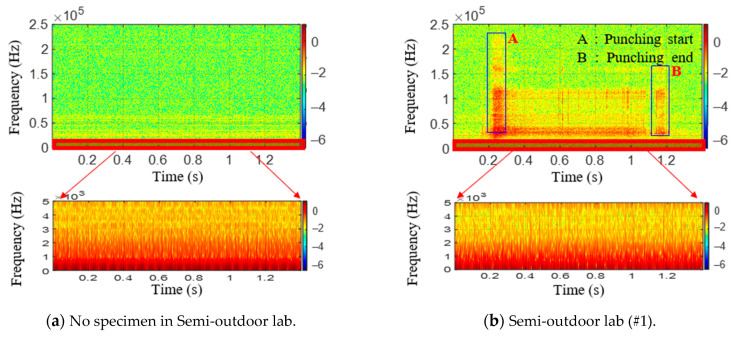

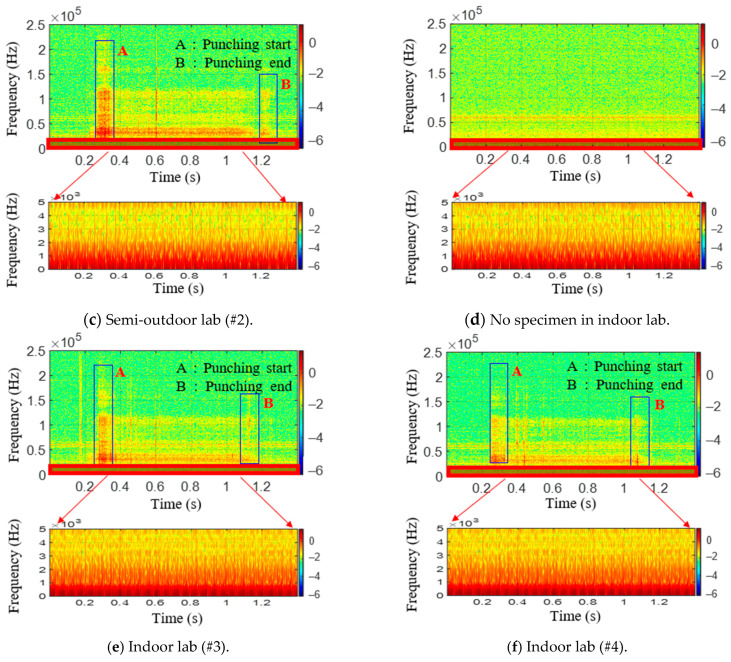
Spectrogram of AE raw data with press process.

Fast Fourier transform (FFT) is applied to the raw signals of testing materials. Figure 11 shows raw AE signal characteristics in the frequency domain. Similar to Figure 9, the signals shown in Figure 10a,b were obtained in a laboratory exposed to the outside environment, and the signals shown in Figure 10c,d were obtained in a laboratory environment. From these figures, the peak frequencies of AE signals in the semi-outdoor labs and indoor labs are 60.3 and 31 kHz, respectively. Short-time Fourier transform (STFT), which is a sequence of Fourier transform of a windowed signal, is applied to the raw signals of the AE sensor [40]. STFT can be visualized by spectrogram. It shows the time-localized frequency information. Similar to Figure 10, the signals shown in Figure 12a–c were obtained in a laboratory exposed to the outside environment, and the signals shown in Figure 12d–f were obtained in a laboratory environment. The results in Figure 12a,d are measured without a specimen during the press process. The vertical and horizontal axes represent frequency and time, respectively. The amplitude of a particular frequency at a particular time is represented by the color of each point in the spectrogram. From the comparison of the results with and without a specimen, the specimen and punch meet at period A (approximately 0.25–0.3 s), and the hole in the specimen expands. Furthermore, the press process ends at period B (approximately 1.1–1.2 s). Due to friction between the punch and specimen, periods A and B show the signal with a wide spectrum (0–2 and 40–200 kHz). Between periods A and B (approximately 0.3–1.1 s), the ripple voltage, noise, and AE signal are measured. 

From the frequency analysis of the measured results in Figure 9 and Figure 12, it is known that the peak frequencies of ripple voltage and noise are under 2 kHz; the peak frequencies of AE signal are near 40–60 kHz, and the magnitudes of ripple voltage and noise are higher than that of the AE signal. Furthermore, the amplitude of the AE signal with a fracture in Figure 12b,e is a little bit larger than that of the AE signal without fracture in Figure 12c,f. Furthermore, the mean frequency (MNF) of the measured results is obtained by the following equation

(8) MNF=∑i=1nfiPi∑i=1nPi

where *f_i_* is frequency value at the frequency bin *i*, *P_i_* is the power spectrum at the frequency bin *i*, and *n* is the length of frequency bin *i*. The mean frequency, which is calculated as the sum of the power spectrum and the frequency divided by the total sum of the power spectrum [41], and standard deviation are also shown in Table 3. As the small amplitude AE signals are not resolved from background noise and ripple voltage, the value of obtained mean frequencies is also small, and its standard deviations are very big. 

## 4. AE Detection Method and Discussions

**Figure 13:**
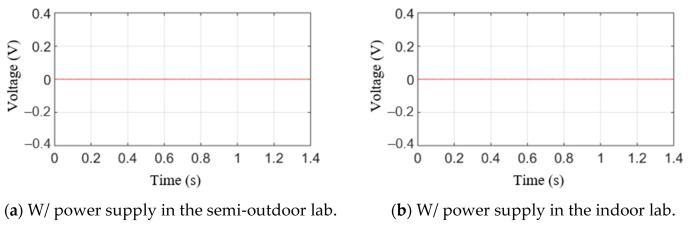
AE filtered signal without press process.

As mentioned earlier, the AE signals at factories where the press operations are performed have a very high signal-to-noise ratio. Therefore, it is very difficult to detect and analyze the AE signals. To detect the AE signals when there were many noises and ripple voltage signals, the KF-RV algorithm was applied to the data from Figure 9, and the results are plotted in Figure 13. By applying the filter to the ripple voltage signal and noise signal in the semi-outdoor lab and indoor lab, the ripple voltage signal and noise signal were averagely reduced by 98.1%. Based on these results, it was found that the proposed KF-RV algorithm effectively attenuates the ripple voltage signal and noise signal.

**Figure 14:**
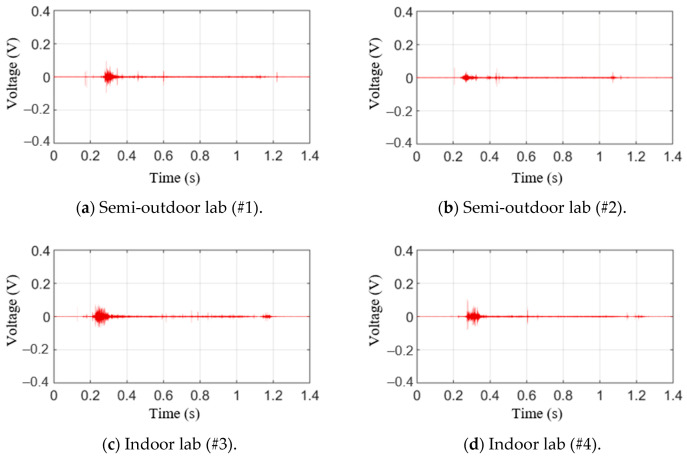
AE filtered signal with press process.

**Figure 15:**
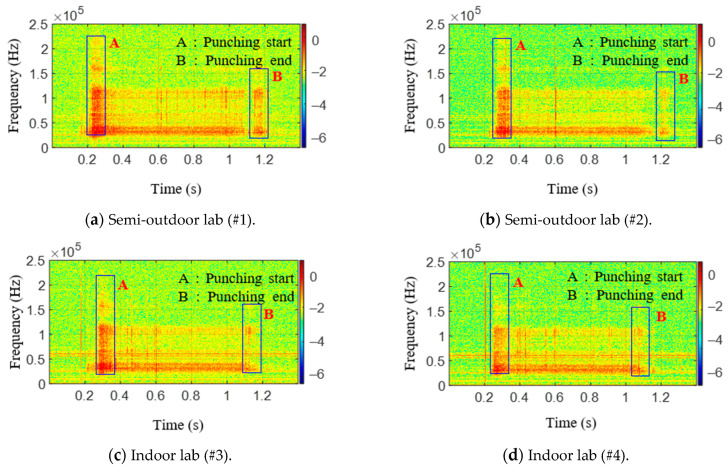
Result of STFT using AE filtered data.

Figure 14 shows the results of applying the KF-RV algorithm to the raw AE signal generated during the press operation. Because the noise and ripple voltage signals have been removed, only the pure AE signal can be seen. In Figure 14a–d, it can be seen that plastic deformation and fractures occurred between 0.2 and 0.4 s primarily due to the press operation. However, it is difficult to determine if a fracture occurred based only on the shape of the graph. Hence, the AE activity was analyzed. As before, the threshold voltage was set, but it was set to 0.035 V, which is half of 0.07 V. Furthermore, the STFT of the filtered results were obtained and plotted in Figure 15. Since there are no signals at a low frequency in Figure 15, it can be confirmed that the noise and ripple voltage signals have been effectively removed. The parameters used in the experiment and the results are shown in Table 4. As shown in Table 4, the test specimens with fractures were identified using the number of ring-down counts. Signals #1 and #3 showed a large number of ring-down counts. When only plastic deformation occurred, which simply expanded the diameter of the hole, AE signals were present, but the number of ring-down counts was low. Furthermore, it can be observed that the mean frequencies of the filtered signals are bigger than that of the raw signal. By removing the noise and ripple voltage signals, the mean frequency of the filtered signal is in the frequency range of the AE event in Table 2. It was determined that this result was obtained because only meaningful AE signals were extracted by setting an appropriate threshold voltage instead of a high threshold voltage due to the ripple voltage signal. 

Thus, the AE signal is generated due to plastic deformation and fracture in sheet metal forming. If the noise and ripple voltage signals can be filtered out appropriately, the occurrence of fracture can be determined by deriving the ring-down count. Based on this outcome, this study confirmed that it is possible to determine the accurate time at which a defect occurs in the product during the sheet metal forming process.

## 5. Conclusions 

In this study, an AE sensor was applied to predict fractures in ductile materials during the sheet metal forming process. Since the AE signal occurs when plastic deformation and fracture of metallic materials occur, the defect of the specimen can be detected in advance. However, the AE signals for ductile materials have a very high signal-to-noise ratio; hence, it is difficult to detect and analyze the AE signals of the ductile materials. So, a Kalman filter with a low Kalman gain was designed to extract only the ripple voltage and noise signal. Subsequently, the AE signal was obtained by subtracting the ripple voltage signal from the original raw signal. In order to confirm the performance of the proposed algorithm, a computer simulation has been conducted, and superior canceling performance was confirmed. By using the KF-RV algorithm, the activity of the extracted AE signal was analyzed using the ring-down count among various AE parameters to determine if there was a fracture in the test specimen. The sheet metal forming test was prepared to evaluate the performance of the proposed detection method. If ripple voltage and noise signals other than the AE signal are filtered out appropriately, the presence or absence of a fracture can be determined using the ring-down count parameter. Based on the test result, this study confirmed that it is possible to determine the accurate time at which a defect occurs in the product during the sheet metal forming process. 

## Figures and Tables

**Table 1:** Comparison of the estimation performances.

	*e_rms_* (%)	*e_max_* (%)
Classical Kalman Filter with Q_1_(0.1)	62.60%	25.49%
Classical Kalman Filter with Q_2_(0.00001)	105.68%	43.02%
KF-RV (Proposed)	34.53%	14.06%

**Table 2:** R6I-AST specifications.

Sensor Specifications					
Peak sensitivity, Ref V/(m/s)	117 dB	Directionality	+/−1.5 dB	Output Drive Impedance	50 Ω
Peak Sensitivity, Ref V/μbar	−23 dB	Gain	40 dB	Temperature Range	−35 to 75 °C
Operation Frequency Range	40–100 kHz	Power Requirements	20–30 VDC @ 25 mA	Shock Limit	500 g
Resonant Frequency, Ref V/(m/s)	55 kHz	Dynamic Range	>87 dB	Dimensions	29 mm OD × 40 mm H
Resonant Frequency, Ref V/μbar	98 kHz	Noise Level (RMS RTI)	<3 μV	Weight	98 g
Face Material	Ceramic	Connector	BNC	Connector Locations	Side

**Table 3:** AE parameters from the raw signal.

	Ring-Down Count	Duration Time	Peak Amplitude	Mean Frequency (Standard Deviation)	Deformation Type
	Unit: EA	Unit: s	Unit: V	Unit: kHz	
#1	152	0.0349	0.1749	7.88 (±19.17)	Plastic deformation and fracture
#2	11	0.184	0.1006	5.16 (±17.3)	Plastic deformation
#3	23	0.0498	0.1139	0.652 (±6.54)	Plastic deformation and fracture
#4	21	0.072	0.1474	0.35 (±4.32)	Plastic deformation

**Table 4:** AE parameters from filtered data.

	Ring-Down Count	Duration Time	Peak Amplitude	Mean Frequency (Standard Deviation)	Deformation Type
	Unit: EA	Unit: s	Unit: V	Unit: kHz	
#1	132	0.03097	0.0967	43.1 (±25.4)	Plastic deformation and fracture
#2	16	0.01892	0.0602	42.3 (±24.4)	Plastic deformation
#3	147	0.051	0.0757	44.3 (±25.9)	Plastic deformation and fracture
#4	69	0.05493	0.1038	45.4 (±27.6)	Plastic deformation
